# Distinct Molecular Pattern-Induced Calcium Signatures Lead to Different Downstream Transcriptional Regulations via AtSR1/CAMTA3

**DOI:** 10.3390/ijms21218163

**Published:** 2020-10-31

**Authors:** Peiguo Yuan, Jeremy B. Jewell, Smrutisanjita Behera, Kiwamu Tanaka, B. W. Poovaiah

**Affiliations:** 1 Laboratory of Molecular Plant Science, Department of Horticulture, Washington State University, Pullman, WA 99164-6414, USA; pomology2010@gmail.com; 2 Department of Plant Pathology, Washington State University, Pullman, WA 99164-6430, USA; jbjewell@wsu.edu (J.B.J.); kiwamu.tanaka@wsu.edu (K.T.); 3 Indian Institute of Chemical Biology, Kolkata, West Bengal 700 032, India; smrutisanjita@gmail.com

**Keywords:** MAMPs, DAMPs, nuclear and cytoplasmic calcium signaling, plant immune response, salicylic acid, jasmonic acid, AtSR1/CAMTA3, flg22, chitin, AtPep1

## Abstract

Plants encrypt the perception of different pathogenic stimuli into specific intracellular calcium (Ca^2+^) signatures and subsequently decrypt the signatures into appropriate downstream responses through various Ca^2+^ sensors. Two microbe-associated molecular patterns (MAMPs), bacterial flg22 and fungal chitin, and one damage-associated molecular pattern (DAMP), AtPep1, were used to study the differential Ca^2+^ signatures in *Arabidopsis* leaves. The results revealed that flg22, chitin, and AtPep1 induced distinct changes in Ca^2+^ dynamics in both the cytosol and nucleus. In addition, Flg22 and chitin upregulated the expression of salicylic acid-related genes, *ICS1* and *EDS1*, whereas AtPep1 upregulated the expression of jasmonic acid-related genes, *JAZ1* and *PDF1.2*, in addition to *ICS1* and *EDS1.* These data demonstrated that distinct Ca^2+^ signatures caused by different molecular patterns in leaf cells lead to specific downstream events. Furthermore, these changes in the expression of defense-related genes were disrupted in a knockout mutant of the *AtSR1/CAMTA3* gene, encoding a calmodulin-binding transcription factor, in which a calmodulin-binding domain on AtSR1 was required for deciphering the Ca^2+^ signatures into downstream transcription events. These observations extend our knowledge regarding unique and intrinsic roles for Ca^2+^ signaling in launching and fine-tuning plant immune response, which are mediated by the AtSR1/CAMTA3 transcription factor.

## 1. Introduction

During plant–pathogen interactions, intracellular calcium (Ca^2+^) transients are known to be an early and necessary event in local and systemic signaling response [1,2]. A specific Ca^2+^ signature is shaped following the perception of microbe-derived molecular patterns (MAMPs) or endogenous damage-associate molecular patterns (DAMPs), by plasma membrane-localized pattern recognition receptors (PRRs) during plant immune response [3,4,5]. For example, the bacterial MAMP flagellin is primarily recognized by a PRR, Flagellin Sensitive 2 (FLS2), in *Arabidopsis* [6]. Recent reports demonstrated that Ca^2+^ responses to flagellin are mediated by plasma membrane-localized Ca^2+^ pumps, autoinhibited Ca^2+^-ATPases (ACA8 and ACA10), potentially by direct interaction with and modulation by FLS2 [7]. Further, the activated FLS2 interacts with botrytis-induced kinase 1 (BIK1), which leads to the accumulation of reactive oxygen species (ROS) by activating respiratory burst oxidase homologs (RBOHs) [8,9]. ROS signal is another trigger of the activation of Ca^2+^ influx channels [1,10,11]. Chitin elicitor receptor kinase 1 (CERK1), associated with lysin motif-containing receptor-like kinases (LYK4 and LYK5), recognizes chitin oligomers [degree of polymerization (dp) of 6–8] as fungal MAMPs [12,13]. CERK1 was reported to activate annexin 1 (ANN1, a Ca^2+^ permeable channel) to trigger specific Ca^2+^ signatures in *Arabidopsis* [14], while in rice, CERK1 was demonstrated to activate RBOH, leading to ROS accumulation, which induced Ca^2+^ influx indirectly [15]. In a similar fashion, perception of DAMPs by PRRs also triggers Ca^2+^ signaling. AtPep1 is a well-documented DAMP recognized by Pep1 receptor 1 and 2 or AtPEPR1 and AtPEPR2 [16,17]. Interestingly, AtPEPR1 has guanylyl cyclase activity [18]. In this case, activated AtPEPR1 generates cGMP, which eventually activates plasma membrane-localized cyclic nucleotide-gated ion channels (CNGCs) to form specific Ca^2+^ signatures [19].

The nucleus is an important organelle for the storage of genetic data. Nuclear Ca^2+^ signals play unique roles during plant–microbe interactions [20]. Recent studies used nuclear-localized Ca^2+^ sensors to test nuclear Ca^2+^ spikes. The Ca^2+^ rise in the nucleus might be generated from the cytoplasm. Many biotic or abiotic stimuli trigger the cytoplasmic Ca^2+^ transients [11,21,22]. Subsequently, nuclear Ca^2+^ signals are induced autonomously through nuclear Ca^2+^ channel or Ca^2+^ pump. In plant–symbiont interaction, lipo-chitooligosaccharides (e.g., Nod factor of rhizobia and Myc factor of arbuscular mycorrhiza fungi) induce nuclear Ca^2+^ transients in the roots, especially in the root hair in most plants, except Brassicas [23]. The induction of nuclear Ca^2+^ transients by MAMPs or DAMPs remains to be determined, although some studies have been carried out in abiotic stresses [23,24].

Cytosolic Ca^2+^ signatures triggered by MAMPs and DAMPs are deciphered into downstream pathways for the appropriate immune response. In plants, there are four main types of Ca^2+^ sensors: calmodulin (CaM), CaM-like proteins (CMLs), calcineurin b-like proteins (CBLs), and Ca^2+^-dependent protein kinases (CDPKs or CPKs) [25,26]. These Ca^2+^ sensors mediate deciphering Ca^2+^ signals to downstream events for plant immune response [2]. CPKs are required to sense and decode MAMP- or DAMP-induced Ca^2+^ signals into phosphorylation events [27,28]. For example, CPK4, CPK5, CPK6, and CPK11 belong to a closely related clade in subgroup I, which decodes Ca^2+^ signatures into the ROS-mediated immune pathway, likely through the phosphorylation of RBOHD [29,30]. Very recently, CPK5 and CPK6 were shown to directly phosphorylate WRKY33, which eventually upregulates camalexin biosynthetic genes to confer plant resistance against a necrotrophic pathogen, *Botrytis cinerea*.

Several Ca^2+^/CaM-regulated transcription factors are involved in decoding nuclear Ca^2+^ signatures [31]. CaM-binding protein 60g (CBP60g), together with SAR (systemic acquired resistance) deficient 1 (SARD1), plays a positive role in salicylic acid (SA)-mediated plant immune pathway, through binding to the promoter of *isochorismate synthase 1 (ICS1)*, which encodes a key enzyme in SA biosynthesis [32,33,34]. In contrast, CBP60a suppresses the accumulation of SA and the expression of *ICS1*, potentially by directly binding to a promotor region of *ICS1* [35]. In addition, CaM-binding transcriptional factor 3 (CAMTA3), also known as AtSR1, plays a negative role in plant immunity [36] by suppressing the expression of both enhanced disease susceptibility 1 (EDS1) and non-race specific disease resistance (NDR1), essential factors leading to the activation of SA synthesis via the involvement of ICS1 [37,38,39,40]. These observations have confirmed that Ca^2+^ serves as a crucial messenger in SA-regulated immune response. In addition, Ca^2+^ signaling plays a key role in jasmonic acid (JA)-mediated immune response. For example, following JA application, the expression of *jasmonate-zim-domain protein 1* (*JAZ1)*, a marker gene of the JA signaling pathway, was partially inhibited in *dnd1/cngc2* mutant plant, which lacks a functional cyclic nucleotide-gated Ca^2+^ permeable channel [41].

Accumulating experimental data indicate that plants are able to encode recognition of different pathogen invasions into specific temporal and spatial Ca^2+^ signatures and decode these Ca^2+^ signatures to launch an appropriate transcriptional expression of immune-related genes [42,43]. However, it is not clearly known how such Ca^2+^ signatures are generated in the nucleus and how these Ca^2+^ signatures convey their message to downstream immune responders. In this study, we used a genetically encoded Ca^2+^ sensor, aequorin (AEQ), to measure the characteristics of Ca^2+^ signals triggered by two MAMPs, flg22 and chitin, and one DAMP, AtPep1. In addition, we investigated nuclear Ca^2+^ signals using the *Arabidopsis* leaf mesophyll protoplast system to transiently express nuclear-tagged Ca^2+^ probe, AEQ-NLS (nuclear localization signal) [44]. We observed the different MAMP- or DAMP-induced unique Ca^2+^ signatures in both the cytosol and nucleus in *Arabidopsis* leaves. We also investigated downstream events of the Ca^2+^ signaling, such as expression of defense-related marker genes regulated by SA and JA. All stimulants substantially induced the expression of SA-regulated genes, but only AtPep1 induced the expression of JA-regulated genes. Interestingly, these upregulations of SA-regulated genes were further exaggerated in a knockout mutant of *AtSR1/CAMTA3*, whereas AtPep1-induced JA-regulated genes were attenuated in the same mutant. Our results demonstrate that MAMPs and DAMPs induce distinct Ca^2+^ signatures that trigger different downstream responses during plant defense/immunity, in which AtSR1 plays a key role in decoding the Ca^2+^ signals into transcriptional reprogramming during plant–microbe interactions.

## 2. Results

### 2.1. MAMP/DAMP-Induced Ca^2+^ Transients in Cytosol

**Figure 1:**
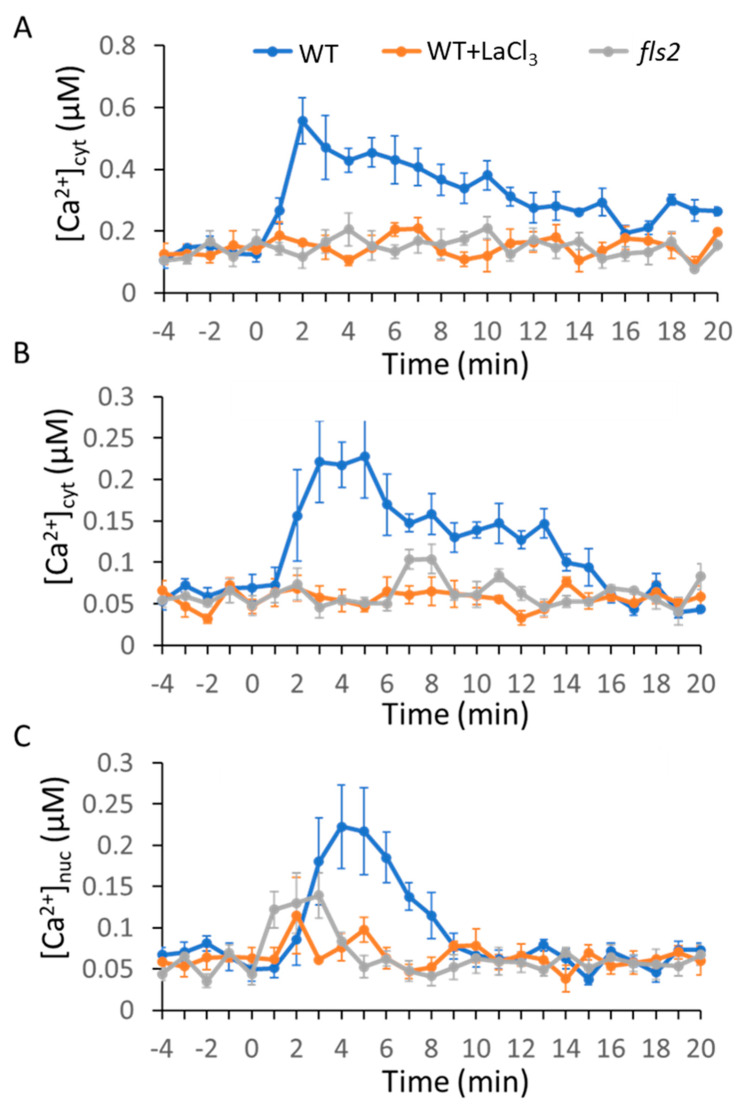
Flg22-induced cytoplasmic or nuclear Ca^2+^ transients in aequorin (AEQ)-expressing *Arabidopsis* plants or transient AEQ-expressing mesophyll leaf protoplasts. Here, 1 µM flg22 was added at time 0 min to wild-type (WT; blue lines), WT with LaCl_3_ (orange lines), or *fls2* mutant plants (grey lines) as noted in (**A**–**C**). (**A**) Leaf samples. (**B**) Leaf mesophyll protoplasts transfected with AEQ. (**C**) Leaf mesophyll protoplasts transfected with AEQ-nuclear localization signal (NLS). The curves shown are averages generated from four biological replicates; leaves for each replicate were taken from different plants. The bioluminescence was measured at 1 min time intervals, and SE was calculated for each mean; SE values are portrayed as error bars.

**Figure 2:**
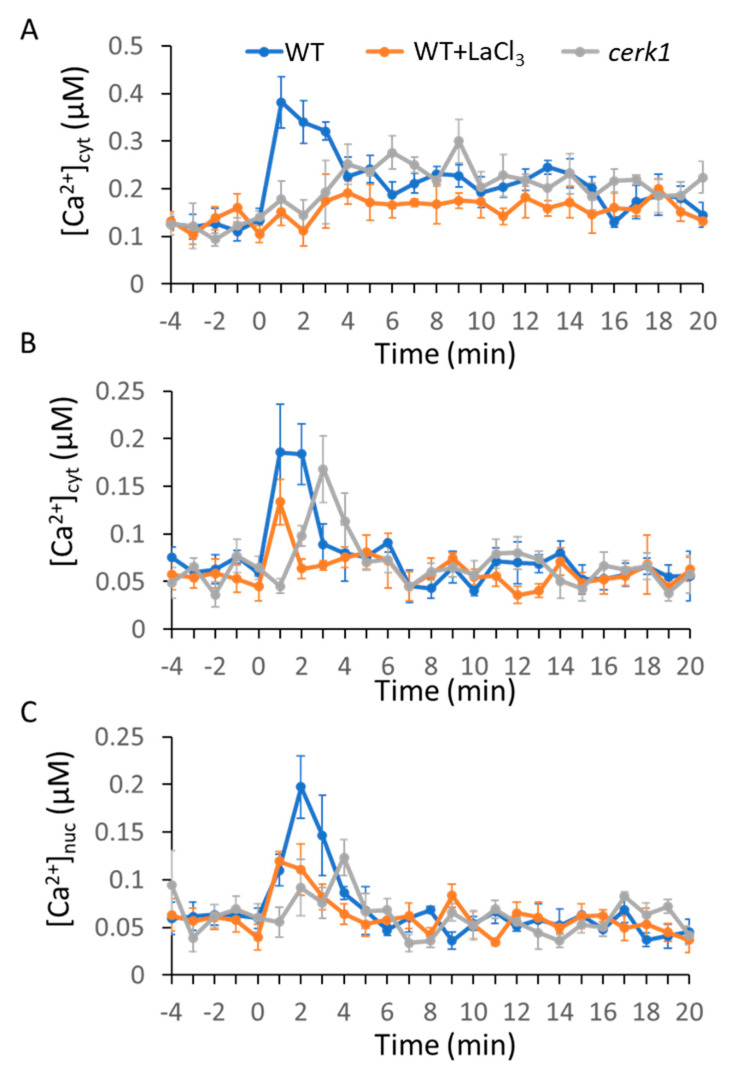
Chitin-induced cytoplasmic or nuclear Ca^2+^ transients in aequorin-expressing *Arabidopsis* plants or transient AEQ-expressing mesophyll leaf protoplasts. Here, 1 µM chitin was added at time 0 min to wild-type (WT; blue lines), WT with La Cl_3_ (orange lines), or *atcerk1* mutant plants (grey lines) as noted in (**A**–**C**). (**A**) The leaf samples. (**B**) Leaf mesophyll protoplasts transfected with AEQ. (**C**) Leaf mesophyll protoplasts transfected with AEQ-NLS. The curves shown are averages generated from four biological replicates; leaves for each replicate were taken from different plants. The bioluminescence was measured at 1 min time intervals, and SE was calculated for each mean; SE values are portrayed as error bars.

**Figure 3:**
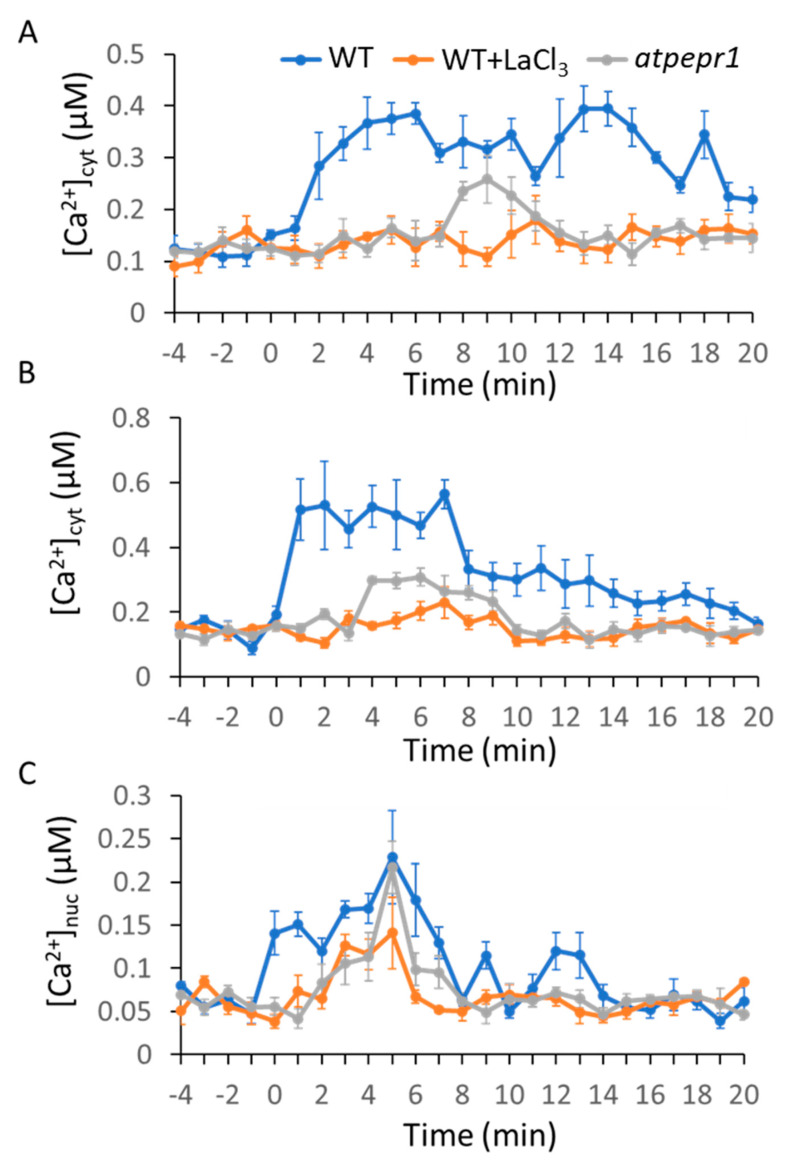
AtPep1-induced cytoplasmic or nuclear Ca^2+^ transients in aequorin-expressing *Arabidopsis* plants or transient AEQ-expressing mesophyll leaf transfected protoplasts. Here, 1 µM AtPep1 was added at time 0 to wild-type (WT; blue lines), WT with LaCl_3_ (orange lines), or *atpepr1* mutant (grey lines) plants as noted in (**A**–**C**). (**A**) The leaf samples. (**B**) Leaf mesophyll protoplasts transfected with AEQ. (**C**) Leaf mesophyll protoplasts transfected with AEQ-NLS. The curves shown are averages generated from four biological replicates; leaves for each replicate were taken from different plants. The bioluminescence was measured at 1 min time intervals, and SE was calculated for each mean; SE values are portrayed as error bars.

To test the effects of MAMP and DAMP on intracellular Ca^2+^ dynamics, we chose two MAMPs, bacterial-derived flg22 and fungal-derived chitin (dp = 8; chitooctaose), and one DAMP, AtPep1. Here, 1 µM flg22 induced a rapid Ca^2+^ transient that reached a maximum level within 2 to 3 min; subsequently, a slow decrease in Ca^2+^ concentration was observed (Figure 1A). Similarly, 1 µM chitin induced a rapid Ca^2+^ transient and the maximum peak was reached within 1 to 2 min, followed by a quick decrease of Ca^2+^ concentration to the resting level in about 15 min (Figure 2A). Interestingly, 1 µM AtPep1 treatment induced more than two Ca^2+^ peaks. At first, there was a slow and gradual rise of Ca^2+^ concentration that reached the maxima within 5 to 6 min. A second peak was observed around 13 min (Figure 3A). We next investigated whether these pattern-triggered Ca^2+^ transients are dependent on Ca^2+^ influx channel. We performed the experiments in the presence of the non-selective Ca^2+^ channel blocker lanthanum (III) chloride (LaCl_3_). The result showed that the clear rise of pattern-triggered cytosolic Ca^2+^ was greatly inhibited when the leaves were pre-treated with 1 mM LaCl_3_ for 1 h (Figure 1A, Figure 2A, and Figure 3A). Furthermore, to test if the molecular pattern-triggered Ca^2+^ transients are required for their specific receptors, the Ca^2+^ reporter AEQ was expressed in each single mutant lacking functional FLS2, CERK1, or AtPEPR1. Our result showed that no significant Ca^2+^ transients were observed in the *fls2, cerk1,* or *Atpepr1* mutant plants (Figure 1A, Figure 2A, and Figure 3A). These observations suggest that various pattern-induced Ca^2+^ signatures in *Arabidopsis* leaves are formed with functional PRRs and Ca^2+^-influx channels. We obtained similar results in the mesophyll protoplasts (Figure 1B, Figure 2B, and Figure 3B), suggesting that most of the signals observed in this study are from the leaf mesophyll cells.

### 2.2. MAMPs/DAMP-Induced Ca^2+^ Transients in the Nucleus

Ca^2+^ transients in the nucleus play a key role during plant–microbe interactions. To determine whether the MAMPs and DAMPs induced Ca^2+^ transients in the nucleus, we used a nuclear localized AEQ (AEQ-NLS) reporter construct and transiently expressed it in leaf mesophyll protoplasts [44]. As shown in Figure 1C, flg22-induced Ca^2+^ transients in the nucleus were initiated at 2 min and reached the maximum peak at 4 to 5 min, followed by a quick drop, and the Ca^2+^ concentration in the nucleus returned to the resting level at 10 min, while the fungal elicitor chitin triggered a very quick Ca^2+^ increase in the nucleus. The Ca^2+^ signals maximize at 2 to 3 min and drop to resting level at 6 min (Figure 2C). AtPep1-induced Ca^2+^ transients in the nucleus started at 0 min (meaning it happened immediately after the addition) and reached the maximum at 5 to 6 min, and then dropped to resting level at 8 min. There were two weaker peaks at 9 min and 12 to 13 min (Figure 3C). As seen in the cytosol, the MAMP- and DAMP-induced Ca^2+^ signatures in the nucleus are required for intact PRRs and Ca^2+^ channels (Figure 1C, Figure 2C, and Figure 3C).

### 2.3. MAMP/DAMP-Induced Transcriptional Reprogramming of SA-Regulated Genes and JA-Regulated Genes

**Figure 4:**
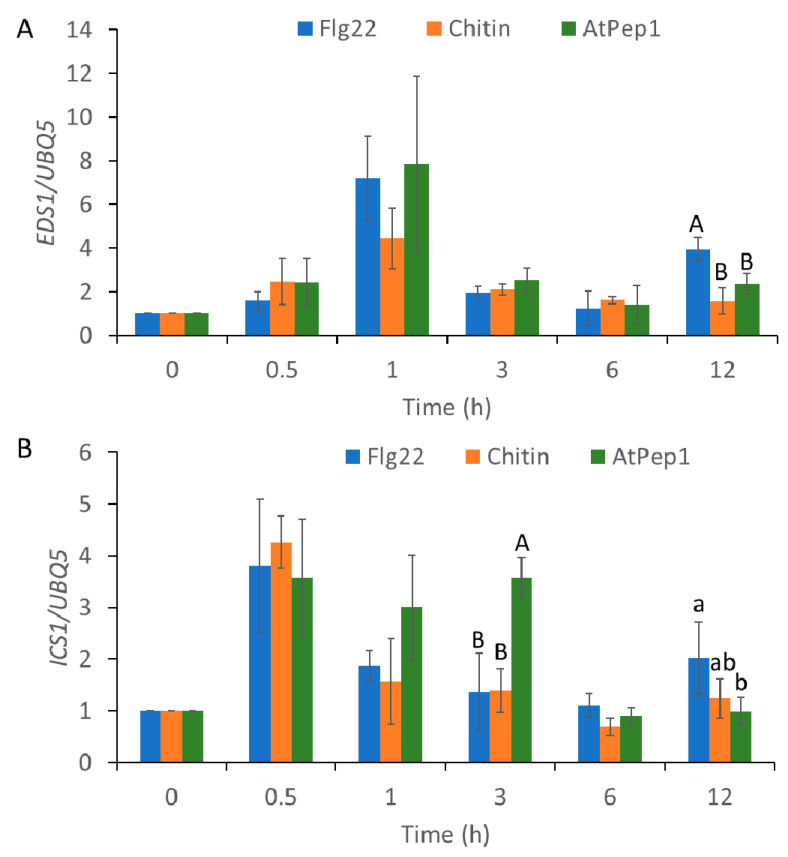
Microbe-associated molecular pattern—(MAMP) and damage-associated molecular pattern (DAMP)-induced transcriptional expression of salicylic acid (SA)-related genes, *EDS1* and *ICS1*. Fold change in *EDS1* (**A**) and *ICS1* (**B**) transcript expression in wild-type (WT) *Arabidopsis* in response to 1 µM of flg22, chitin, and AtPep1 at 0, 0.5, 1, 3, 6, and 12 h after the start of treatment. Total RNA samples were prepared from leaves. SA-related gene expression was normalized to that of the *UBQ5* gene. Values were means ± SD of three biological replicates. Different letters indicate statistically significant differences among treatments analyzed by one-way analysis of variance (ANOVA) (*p* < 0.05) with Tukey test.

SA is an important defense hormone and is involved in MAMPs- or DAMPs-induced immune response in plants. To study the MAMPs- or DAMPs-induced transcriptional reprogramming of SA-related genes, *EDS1* and *ICS1* were selected. All the stimulants, flg22, chitin, and AtPep1, triggered similar fold changes and temporal trends in *EDS1* gene expressions (Figure 4A); its upregulation started at 0.5 h after treatment, and returned to the initial level at 3 to 12 h after treatment. The expression of *ICS1* gene was induced by flg22 and chitin at 0.5 h after inoculation, and returned to the initial level at 1 h after inoculation (Figure 4B). In contrast, AtPep1 significantly upregulated *ICS1* at 0.5 h after inoculation; the upregulation of its expression remained at higher levels at 1 and 3 h after inoculation. These results indicate that AtPep1 differentially reprograms SA-related genes in comparison with flg22 and chitin.

**Figure 5:**
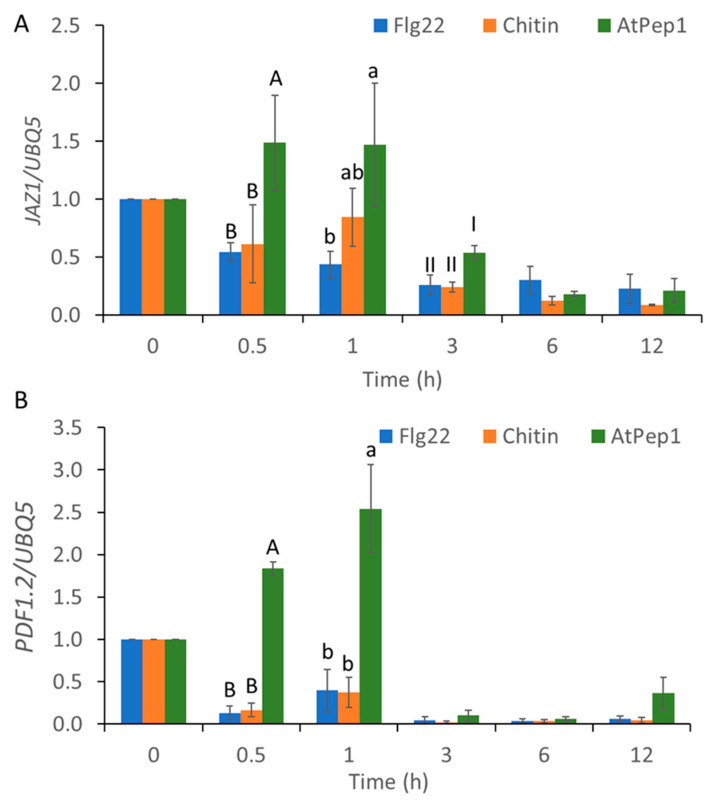
MAMP- and DAMP-induced transcriptional expression of jasmonic acid (JA)-related genes, *JAZ1* and *PDF1.2*. Fold change in *JAZ1* (**A**) and *PDF1.2* (**B**) transcript expression in wild-type (WT) *Arabidopsis* in response to 1 µM of flg22, chitin, and AtPep1 at 0, 0.5, 1, 3, 6, and 12 h after the start of treatment. Total RNA samples were prepared from leaves. JA-related gene expression was normalized to that of the *UBQ5* gene. Values were means ± SD of three biological replicates. Different letters indicated statistically significant differences among treatments analyzed by one-way ANOVA (*p* < 0.05) with Tukey test.

We next examined the expression of JA-responsive gene expression. As shown in Figure 5, expressions of *JAZ1* and *plant defensin 1.2* (*PDF1.2)* were induced by the application of AtPep1 at 0.5 and 1 h, but not flg22 or chitin (Figure 5A,B). These results suggest that AtPep1 induces downstream gene expressions in a different manner in comparison with flg22 and chitin. This difference perhaps can be attributed to unique and distinct Ca^2+^ signaling caused by AtPep1.

To test whether transcriptional reprogramming of defense-related genes induced by MAMPs or DAMPs requires Ca^2+^ influx channels, we performed the experiments using Ca^2+^ channel blocker LaCl_3_ and the calcium chelator, ethylene glycol-bis(β-aminoethyl ether)-N,N,N′,N′-tetraacetic acid (EGTA). To this end, the leaf discs were pretreated with 100 µM LaCl_3_ or 5 mM EGTA for 30 min, and then treated with flg22, chitin, or AtPep1 for 0.5 and 1 h. As shown in Supplementary Figures S1 and S2, the results indicated that MAMP- or DAMP-induced transcriptional reprograming of the defense-related genes requires Ca^2+^ influx.

### 2.4. AtSR1/CAMTA3 Mediates Pattern-Triggered Transcriptional Reprograming of SA- and JA-Regulated Genes

**Figure 6:**
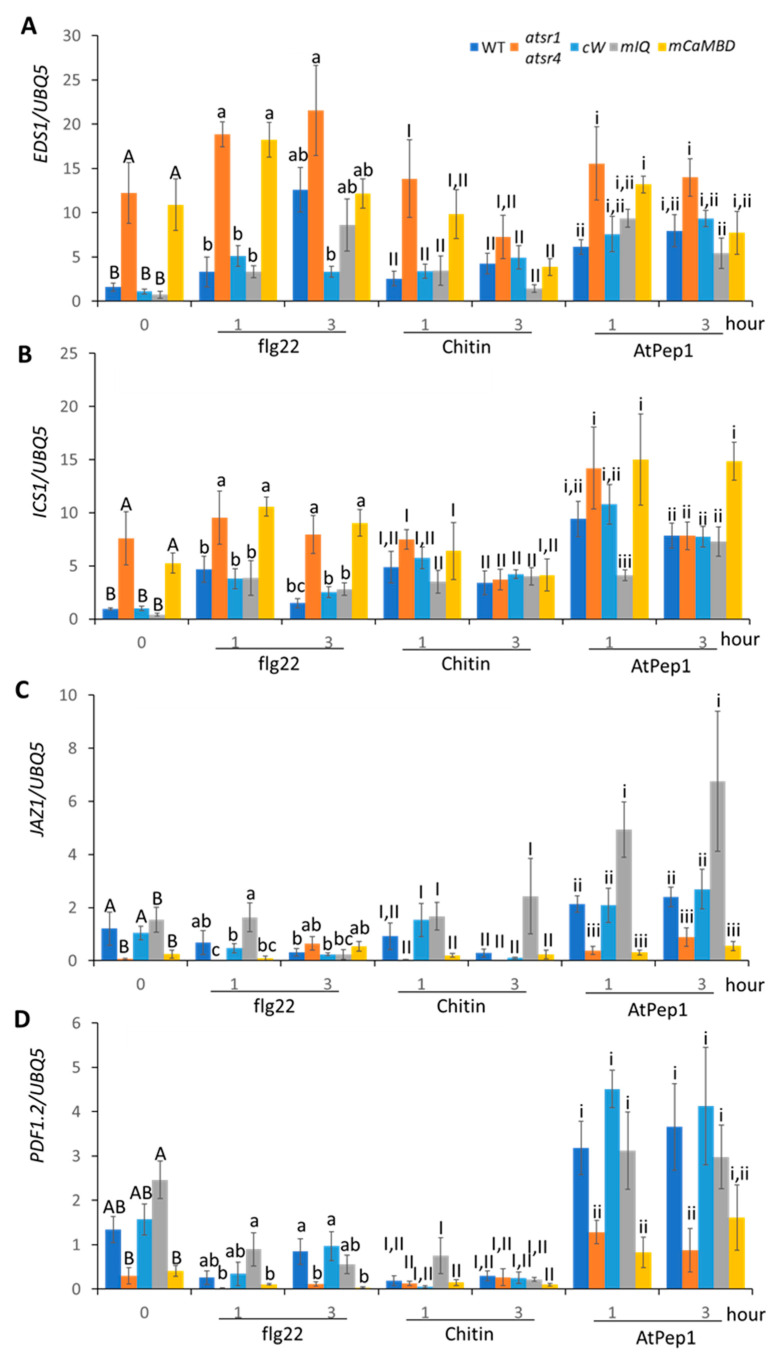
MAMP- and DAMP-induced transcriptional expression of SA- or JA-related genes in *atsr1* and its complementation lines. Fold change in transcriptional expressions of (**A**) *EDS1*, (**B**) *ICS1*, (**C**) *JAZ1*, and (**D**) *PDF1.2* was shown in wild-type (WT), *atsr1 atsr4*, and its complementation lines. Transgenes of complementation lines are intact *AtSR1* gene (*cW*) and mutant *AtSR1* gene defective either of IQ motif (*AtSR1^A855V^ = mIQ*) or CaMBD (*AtSR1^K907E^ = mCaMBD*). The *Arabidopsis* leaves were treated with 1 µM of flg22, chitin, and AtPep1 for 1 or 3 h. The gene expression was normalized to that of the *UBQ5* gene. Values were means ± SD of three biological replicates. Different letters indicated statistically significant differences among treatments analyzed by one-way ANOVA (*p* < 0.05) with Tukey test.

It is reported that Ca^2+^, CaM, and CaM-binding transcriptional factors work together to amplify specific Ca^2+^ signals to regulate gene expressions [42]. In addition, AtSR1 is a well-studied CaM-binding transcription factor and includes several CaM-binding domains [37]. To test whether MAMP/DAMP-induced transcriptional reprogramming of defense-related genes is mediated by Ca^2+^ sensors, we measured the expression of SA-regulated genes and JA-regulated genes in a double knockout mutant of AtSR1 and AtSR4 (alias CAMTA3 and CAMTA2, respectively). The results showed that upregulation of SA-regulated genes (by flg22, chitin, and AtPep1) was further exaggerated in the double mutant *atsr1 atsr4* in comparison with those in the wild-type (WT) plants (Figure 6A,B), while upregulation of JA-regulated genes (by AtPep1) was abolished in the double mutant, suggesting that CaM-binding transcription factors, AtSR1 and/or AtSR4, are involved in both transcriptional suppression and activation. We further tested if these transcriptional changes were dependent on Ca^2+^ sensing in AtSR1. To this end, we used three complementation transgenic lines in the *atsr1 atsr4* mutant, where the transgenes used were intact *AtSR1* gene (cW) and mutant *AtSR1* gene defective of either CaM binding sites, i.e., IQ motif (*AtSR1^A855V^ = mIQ* in the figure) or CaMBD (*AtSR1^K907E^ = mCAMBD* in the figure). As shown in Figure 6, the intact AtSR1 gene (cW) fully complemented the phenotype of mis-regulation in expression of SA- and JA-regulated genes in *atsr1 atsr4*, suggesting that single AtSR1 transgene is enough to overcome the defect of not only AtSR1 itself, but also AtSR4. Interestingly, our data demonstrated that only AtSR1^K907E^ did not complement the *arsr1 atsr4* mutant phenotype (Figure 6), which shows exaggerated expression of SA-regulated genes and abolished the expression of JA-regulated genes. These results suggest that the fully functioning AtSR1 is essential for the transcriptional reprogramming of defense-related genes in response to MAMP/DAMP, where the CaMBDs are required for deciphering the specific Ca^2+^ signals into downstream transcription events.

## 3. Discussion

In this study, we investigated the relationships between molecular pattern-induced specific Ca^2+^ signatures and transcriptional reprograming of defense-related genes as evidenced by the downstream immune responses after deciphering of the Ca^2+^ signatures. This study extends our knowledge about the role of Ca^2+^ signal in plant immune response as well as encoding and decoding of Ca^2+^ signatures to launch and establish proper immune response during plant–microbe interactions.

It has been reported that most of the environmental cues induced a transient rise of intercellular Ca^2+^ concentration in plants [45,46]. Increasing attention has been paid to the recognition of different cues by specific receptor(s), leading to distinct calcium transients. However, how plants decode the calcium transients to establish the proper transcriptional responses to specific cues is not clear. Our study confirmed a unique calcium signature generated by each molecular pattern. Both the bacterial MAMP flg22 and the fungal MAMP chitin induced a transient Ca^2+^ signature in leaf cells, although the duration and amplitude of flg22-triggered Ca^2+^ signatures were different from those of chitin-triggered Ca^2+^ signature (Figure 1 and Figure 2). Ca^2+^ signals generated by MAMPs have been characterized in previous studies [18,47,48,49,50]; our observations, together with previous studies, suggested that plants encoded the perception of different MAMPs into distinct Ca^2+^ signatures. In addition, the peptide DAMP AtPep1 induced a specific Ca^2+^ signature in both the cytoplasm and nucleus in the mesophyll cells (Figure 3). These observations suggest that the plant cells recognize different molecular patterns by the individual PRRs that encode into specific Ca^2+^ signatures via Ca^2+^ influx channels. FLS2 suppresses the activation of AtACA8/ACA10, CERK1 activates ANN1, while AtPEPR1 activates CNGCs [51,52,53,54]. Another molecular mechanism by which these PRRs might activate Ca^2+^ channels or pumps localized in specific calcium stores cannot be excluded, although direct evidence is still missing [55,56,57]. Recognition of both flg22- and chitin-triggered ROS-mediated Ca^2+^ transients occur through the activation of RBOH; however, FLS2 and CERK1 activate RBOH through different pathways. FLS2, together with BAK1 and BIK1, phosphorylated and activated RBOH, while in *rice*, CERK1 activated RBOH through OsRac/Rop GTPases [58,59,60]. These differences might be reflected in the formation of distinct Ca^2+^ signatures upon recognition of various molecular patterns.

Our study further revealed that unique Ca^2+^ signatures in the nucleus were induced by each molecular pattern we tested. Flg22 induced a nuclear Ca^2+^ signature similar to the cytoplasmic Ca^2+^ signature. Interestingly, there was a quick decrease in the nuclear Ca^2+^ signature (Figure 1B,C). Chitin-triggered nuclear Ca^2+^ signature was also similar to that in the cytoplasm in WT plants (Figure 2B,C). However, chitin-triggered Ca^2+^ transients in the cytoplasm and the nucleus were compromised in *cerk1* mutant plants (Figure 2B,C). AtPep1 induced prolonged Ca^2+^ transients in both the nucleus and cytoplasm, but the amplitude of the rise of Ca^2+^ was lower in the nucleus than in the cytoplasm (Figure 3B,C). Given that there are Ca^2+^ sensors in the nucleus (e.g., AtSR1, CBP60g), this Ca^2+^ dynamic in the nucleus is important in causing specific transcriptional programming. The origin of nuclear Ca^2+^ spikes has been subjected to debate as to whether nuclear Ca^2+^ signals are induced by cytoplasmic Ca^2+^ increase or if nuclear localization Ca^2+^ channels and/or pumps are used. Similar results were observed in dihydrosphingosine-induced Ca^2+^ spike in tobacco BY-2 cells [61].

In previous studies, more attention was paid to the molecular mechanism of decoding Ca^2+^ signatures into downstream pathways [62]. A new mathematical model was developed to predict the decoding of Ca^2+^ signatures at the transcriptional level [43], which revealed details in the relationship between Ca^2+^ signatures and phytohormone-related immune response. In this study, we observed that the transient Ca^2+^ signatures, triggered by flg22 and chitin, were decoded into SA-related genes and induced a similar fold change and temporal trend of SA-related gene expression. On the other hand, the different oscillatory Ca^2+^ signature triggered by AtPep1 induced a prolonged expression of the SA-related gene, *ICS1* (Figure 4). Moreover, AtPep1 induced JA-regulated genes, but flg22 and chitin did not (Figure 5), and a previous study found that the induction of PDF1.2 was not tested 3 h past the application of flg22 [63]. However, other studies have revealed that flg22 also induced the expression of JA-related genes [64,65]. One possible explanation is that flg22 induced the expression of JA-related genes in root or the application of flg22 in root activated induced-systemic resistance and primed plant immune response by transcriptional reprogramming of JA-related genes. In our study, all observations were conducted using leaf tissue. These results suggest that the differences in Ca^2+^ signatures impact the downstream transcriptional reprograming of the defense-related genes, for which different decoding processes are likely involved.

AtSR1/CAMTA3 is a transcription factor and a Ca^2+^ sensor, which translates Ca^2+^ signatures directly into activation or suppression of specific gene transcriptions. Our data revealed that AtSR1 was involved in the suppression of SA-regulated genes, for which intact CaMBD was required. This result suggests that AtSR1 is activated by sensing the Ca^2+^ transients with its CaMBD and then binding to the cis-element “vCGCGb” on the promoter region of the SA-regulated genes to suppress their expressions, although how other Ca^2+^ sensors mediate activation of SA-regulated genes remains unclear in this case. Interestingly, AtSR1 was involved in the activation of JA-regulated genes, for which CaMBD was again important to its function. In this case, it still remains unclear how AtSR1 directly regulates the promoter activities of JA-regulated genes. Although further research is required to understand comprehensively, our observations of AtSR1 function may shed light on how the Ca^2+^ sensors decode different Ca^2+^ signatures and in turn regulate transcriptional reprograming for plant defense responses.

Recently, an interesting model was provided to explain how plants interpreted specific calcium signatures to generate specific transcriptional profiles in response to various cues, through Ca^2+^-CaM-TF interaction [42]. The model suggests that the induced Ca^2+^ signal was propagated by Ca^2+^-CaM-TFs in a non-linear way, which enables plants to effectively distinguish the kinetics of different calcium signatures induced by different MAMPs or DAMPs; subsequently, the number of active TFs and their DNA-binding affinity resulted in distinct gene expressions to establish a specific response in plant cells [42].

## 4. Materials and Methods

### 4.1. Plant Material and Growth Conditions

The *Arabidopsis* lines used in this study are wild-type (WT) Columbia (Col-0) and three transgenic lines (*fls2* [44], *cerk1* [66], and *pepr1* [18]), as well as the WT and the three mutant lines carrying the Ca^2+^ probe AEQ-expressing transgenic plants. WT and the four complementation lines in *atsr1 atsr4* background, i.e., *cW*, *mIQ* (*AtSR1^A855V^*), and *mCaMBD* (*AtSR1^K907E^*), were used for testing the gene expression [67]. Seeds were surface sterilized with 1/3 diluted bleach for 10 min and washed five times with sterilized water. The sterilized seeds were put onto half-strength Murashige and Skoog (MS) medium (Caisson Laboratories Inc.) containing 1% sucrose and 10 mM 2-(N-morpholino) ethanesulfonic acid (MES) pH 5.8 with KOH, at 4 °C in the dark for 3 days, and geminated in a growth chamber under a 12 h photoperiod/12 h dark light condition at 20 °C. One-week-old seedlings were transferred to pots containing soil mix. Plants were maintained in a growth chamber under a 12 h photoperiod/12 h dark light condition at 20 °C and plants were watered as needed. Leaf samples from four-week-old plants were used in all the experiments.

### 4.2. Calcium Measurements in Leaf Discs

The calcium spikes in leaves were measured with an aequorin-based calcium probe [50,68]. The leaf discs (5 mm) were cut from four-week-old WT or the three mutated *Arabidopsis* lines carrying AEQ, as described above. The leaf discs were placed into a black 24-well plate (three leaf discs in one well) in 1 mL Ca^2+^ imaging buffer (5 mM KCl, 10 mM MES, 10 mM CaCl_2_, pH 5.8 with KOH) with 5 μM coelenterazine (NanoLight Technolgies). The plate was draw vacuumed for 10 min twice. Then, the leaf discs were incubated with coelenterazine solution overnight in the dark at room temperature for reconstitution of AEQ. The following day, the coelenterazine solution was removed by pipette and leaf discs were washed with Ca^2+^ imaging buffer twice. The Ca^2+^-based bioluminescence was quantified in luminometer (Fluoroskan Ascent FL 2.0) for 5 min as baseline. Data were collected for 1 s every 1 min. An equal volume of double-strength pathogen elicitors was added and quantified for 20 min, as L. The total remaining Ca^2+^ in each microplate well was discharged by treatment with equal volumes of 2 M CaCl_2_ in 20% ethanol with 2% NP-40 to discharge the reconstituted remaining AEQ, as L_max_. Ca^2+^ concentration in plant cells was calculated as described previously [50]; the equation is as follows: [Ca^2+^]_cyt_ (nM) = [X + (X x 55) − 1]/(1 − X)/0.02, where X = (L/L_max_)^1/3^.

### 4.3. Calcium Measurements in Arabidopsis Leaf Protoplasts

Here, 5 × 10^4^/mL *Arabidopsis* protoplasts cells were isolated from three-week-old WT or three mutant plants (*fls2, cerk1 and pepr1*) grown at 22 °C, and the cells were transfected with 10 µg of AEQ-red fluorescent protein (RFP) or AEQ-NLS plasmids with the PEG-mediated transfection method, as described previously [44]. Samples were incubated overnight at room temperature with light in WI buffer (4 mM MES, 0.5 M mannitol, 20 mM KCl, pH 5.7) with 2 mM CaCl_2_. The following morning, the protoplasts were harvested at 100× *g* with centrifuge and washed with fresh Ca^2+^-containing WI buffer once; then, the cells were centrifuged and resuspended with the same WI buffer with a 5 µM final concentration of coelenterazine. Then, 80 µL *Arabidopsis* protoplasts cells were put into wells of black 96-well microplates (Perkin Elmer, Waltham, MA, USA) and incubated for 1 h in the dark before starting the Ca^2+^ measurements. The MAMPs- or DAMPs-triggered Ca^2+^ signals were tested as described above. To rule out variations in transfection efficiencies, RFP signals were measured as an internal control.

### 4.4. Chemicals, Buffers, and Elicitors

Stock solutions of 1 mM flg22, 1 mM chitin, 1 mM AtPep1, and 1 M LaCl_3_ were dissolved in Ca^2+^ imaging solution (5 mM KCl, 10 mM MES, 10 mM CaCl_2_, pH 5.7) as stock solution. For immune-stimulus treatment, the working solutions were freshly prepared with stock solution by diluting 1:1000 in Ca^2+^ imaging solution. Ca^2+^ imaging was performed as described above.

### 4.5. RNA Preparation and Real-Time PCR Analysis

Five or six leaf discs from four-week-old *Arabidopsis* plants were transferred into a single well of a 24-well plate and incubated in half-strength MS liquid medium (pH 5.8) overnight in a growth chamber. Control and treated leaf discs were collected and flash frozen in liquid nitrogen (N_2_). The frozen tissues were ground to powder in 1.5 mL microfuge. Total RNA was prepared using TRIzol Reagent (Invitrogen) followed by DNase-I (Roche) treatment. Two micrograms of total RNA were used to synthesize cDNA with random primer and oligo (dT) primer using an advantage RT-for-PCR kit (Clontech, Mountain View, USA). The cDNA was diluted five times and 1 μL/reaction (10 μL) was used as a template. Real-time PCR was performed using an Eppendorf single-color real-time PCR detection system with SYBR Green Supermix (Bio-Rad). Target gene expression levels were normalized to the *AtUBQ5* (AT3G62250). The expression level in WT control was considered to be 1. A minimum of two technical replicates and three biological replicates were used for each experiment. All primers for qRT-PCR are listed in Table S1.

### 4.6. Statistical Analyses

Statistical analyses were performed using JMP software (version 15, SAS Institute, Cary, NC, USA). For experimental data, at least three independent repetitions were performed. The average value from all of the independent repetitions is shown in the figures. One-way ANOVA Tukey’s test was used followed by Tukey honest difference (HSD) test for statistical analysis. Different letters above the columns were used to indicate differences that are statistically significant (*p* < 0.05).

## Supplementary Materials

The following are available online at https://www.mdpi.com/1422-0067/21/21/8163/s1, Figure S1: MAMPs- and DAMPs-induced transcriptional expression of SA-related gene, ICS1, was blocked by Ca^2+^ channel blockers (La^3+^) and Ca^2+^ chelator (EGTA), Figure S2: AtPep1-induced transcriptional expression of JA-related genes, JAZ1 and PDF1.2, was blocked by Ca^2+^ channel blockers, La^3+^ and Ca^2+^ chelator EGTA, Table S1: List of primers for qRT-PCR for SA or JA related genes.
