# Supplementary material for: Distinct Molecular Pattern-Induced Calcium Signatures Lead to Different Downstream Transcriptional Regulations via AtSR1/CAMTA3
Source: Int J Mol Sci. 2020 Oct 31;21(21):8163. doi: 10.3390/ijms21218163 (PMC7662696; doi:10.3390/ijms21218163)
Supplement: Supplementary file 1 [file ijms-21-08163-s001.pdf]

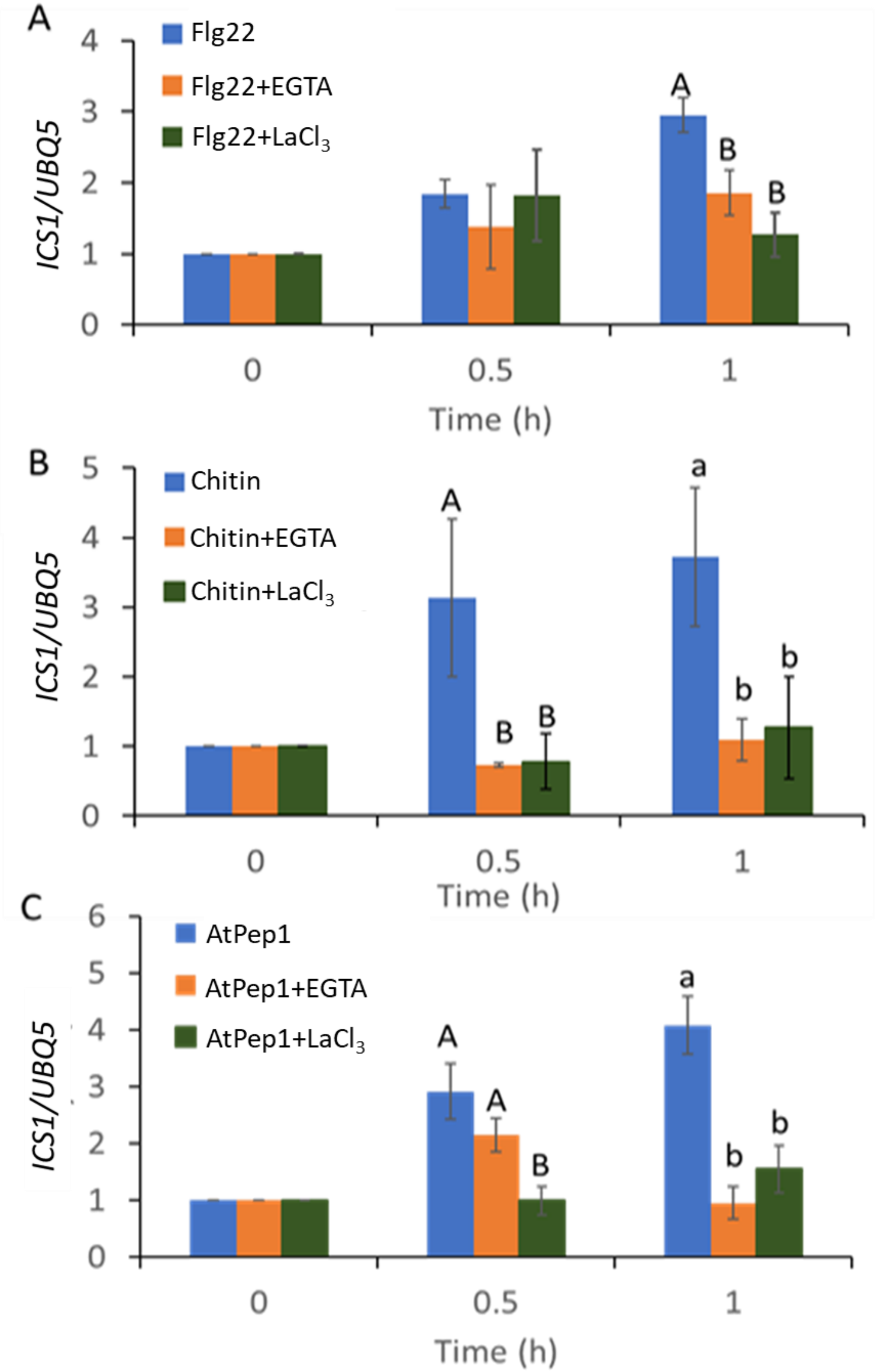

Figure S1. MAMPs- and DAMPs-induced transcriptional expression of SA-related genes, *ICS1*, was blocked by Ca^{2+} channel blockers (La^{3+}) and Ca^{2+} chelator (EGTA). (**A**) Fold change in *ICS1* transcript expression in wild-type *Arabidopsis thaliana* in response to 1μM flg22, (**B**) 1 μM 8-mer

chitin or (**C**) 1 μM AtPep1 with 1mM EGTA, or with 100 μM $LaCl_3$ at 0, 0.5, 1 h after start of treatment. Total RNA samples were prepared from leaves. *ICS1* gene expression was normalized to that of the *UBQ5* gene. Values were means ± SD of three biological replicates. Different letters indicated statistically significant differences among treatments analyzed by one-way ANOVA ($p < 0.05$) with Tukey test.

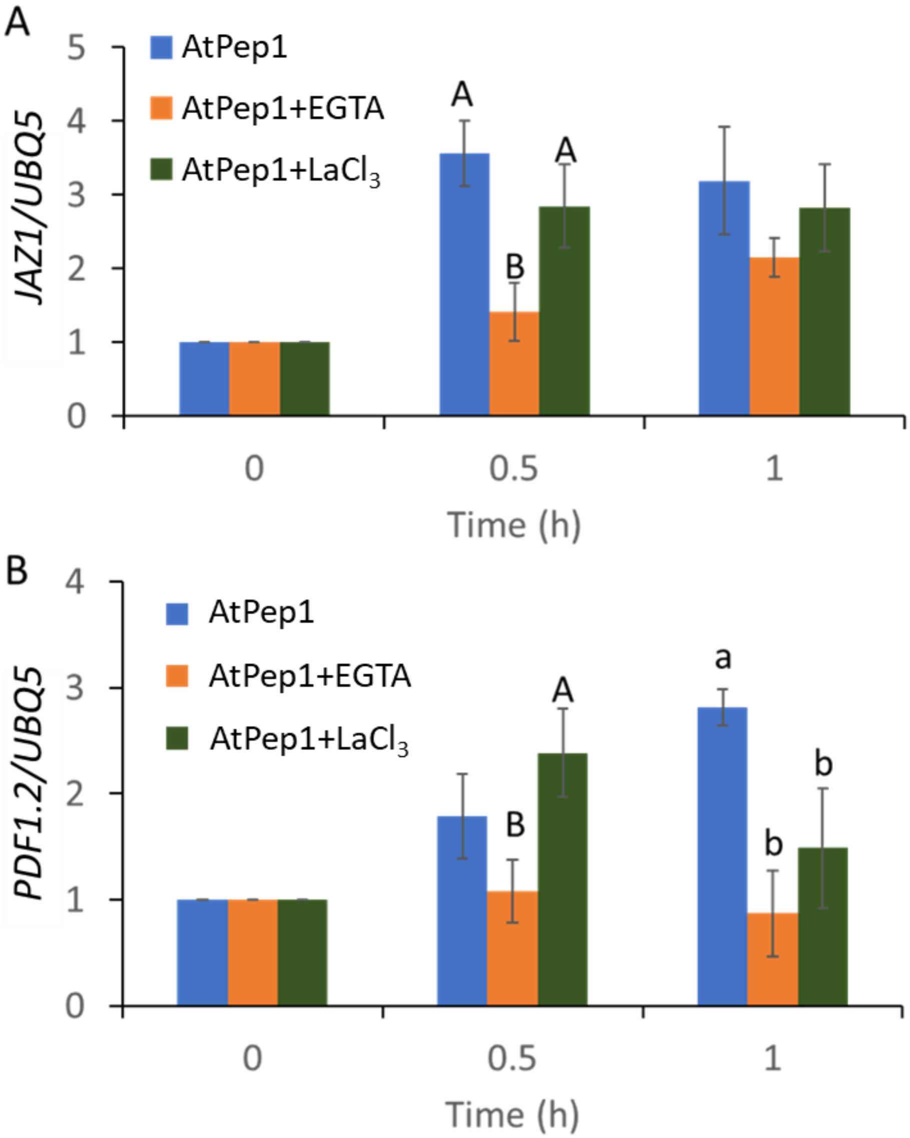

Figure S2. AtPep1-induced transcriptional expression of JA-related genes, *JAZ1* and *PDF1.2*, was blocked by Ca^{2+} channel blockers, La^{3+} and Ca^{2+} chelator EGTA. Fold change in (**A**) *JAZ1* or (**B**) *PDF1.2* transcript expression in wild-type *Arabidopsis thaliana* in response to 1 μM AtPep1 with 1mM EGTA, or 100 μM $LaCl_3$ at 0, 0.5, 1 h after start of treatment. Total RNA samples were prepared from leaves. *JAZ1* and *PDF1.2* gene expressions were normalized to that of the *UBQ5* gene. Values were means ± SD of three biological replicates. Different letters indicated statistically significant differences among treatments analyzed by one-way ANOVA ($p < 0.05$) with Tukey test.

Table S1. List of primers for qRT-PCR for SA or JA related genes

Gene Name/ID	Forward Primer	Reverse Primer
UBQ5	TACCCTCGCCGACTACAACATCC	CTTCTTCCTCTTCTTAGCACCACCAC
EDS1	ACAGAGAAGAGATTGGTTGCAGTGAA	ATTCTTGTTCATCGCTGGCTTGAGA
ICS1	TAATCTGGTTAGCGTTGCTGGTATCG	CCTTCTGATGGATCTCCAATCGTCAT
JAZ1 (1)	GAGCAAAGGCACCGCTAATA	TGCGATAGTAGCGATGTTGC
PDF1.2 (2)	TGTTCTCTTTGCTGCTTTCGACGC	TGTGTGCTGGGAAGACATAGTTGC

References

1. **Grunewald W, Vanholme B, Pauwels L, Plovie E, Inzé D, Gheysen G, Goossens A. 2009.** Expression of the Arabidopsis jasmonate signalling repressor JAZ1/TIFY10A is stimulated by auxin. *EMBO Reports* **10**(8): 923-928.

2. **Niu Y, Figueroa P, Browse J. 2011.** Characterization of JAZ-interacting bHLH transcription factors that regulate jasmonate responses in Arabidopsis. *Journal of Experimental Botany* **62**(6): 2143-2154.
